# Clinical and Laboratory Findings of COVID-19 in High-Altitude Inhabitants of Saudi Arabia

**DOI:** 10.3389/fmed.2021.670195

**Published:** 2021-05-12

**Authors:** Mostafa Abdelsalam, Raad M. M. Althaqafi, Sara A. Assiri, Taghreed M. Althagafi, Saleh M. Althagafi, Ahmed Y. Fouda, Ahmed Ramadan, Mohammed Rabah, Reham M. Ahmed, Zein S. Ibrahim, Dalal M. Nemenqani, Ahmed N. Alghamdi, Daifullah Al Aboud, Ahmed S. Abdel-Moneim, Adnan A. Alsulaimani

**Affiliations:** ^1^Alameen Hospital, Taif, Saudi Arabia; ^2^Mansoura Nephrology and Dialysis Unit, Internal Medicine Department, College of Medicine, Mansoura University, Mansoura, Egypt; ^3^College of Medicine, Taif University, Taif, Saudi Arabia; ^4^Internal Medicine Department, King Abdulaziz Hospital, Jeddah, Saudi Arabia; ^5^General Department of Medical Services, Security Forces Hospital, Mecca, Saudi Arabia; ^6^Anesthesiology Department, Faculty of Medicine, Ain Shams University, Cairo, Egypt; ^7^Radiology Department, Faculty of Medicine, Cairo University, Giza, Egypt; ^8^Albbassia Chest Hospital, Cairo, Egypt; ^9^Department of Physiology, Faculty of Veterinary Medicine, Kafrelsheikh University, Kafrelsheikh, Egypt

**Keywords:** COVID–19, SARS-CoV-2, high-altitude, crp, CO-RADS classification, d-dimer

## Abstract

**Background:** SARS-CoV-2, the causative agent of COVID-19, continues to cause a worldwide pandemic, with more than 147 million being affected globally as of this writing. People's responses to COVID-19 range from asymptomatic to severe, and the disease is sometimes fatal. Its severity is affected by different factors and comorbidities of the infected patients. Living at a high altitude could be another factor that affects the severity of the disease in infected patients.

**Methods:** In the present study, we have analyzed the clinical, laboratory, and radiological findings of COVID-19-infected patients in Taif, a high-altitude region of Saudi Arabia. In addition, we compared matched diseased subjects to those living at sea level. We hypothesized that people living in high-altitude locations are prone to develop a more severe form of COVID-19 than those living at sea level.

**Results:** Age and a high Charlson comorbidity score were associated with increased numbers of intensive care unit (ICU) admissions and mortality among COVID-19 patients. These ICU admissions and fatalities were found mainly in patients with comorbidities. Rates of leukocytosis, neutrophilia, higher D-dimer, ferritin, and highly sensitive C-reactive protein (CRP) were significantly higher in ICU patients. CRP was the most independent of the laboratory biomarkers found to be potential predictors of death. COVID-19 patients who live at higher altitude developed a less severe form of the disease and had a lower mortality rate, in comparison to matched subjects living at sea level.

**Conclusion:** CRP and Charlson comorbidity scores can be considered predictive of disease severity. People living at higher altitudes developed less severe forms of COVID-19 disease than those living at sea level, due to a not-yet-known mechanism.

## Introduction

Humans worldwide have been besieged by the relentless spread of the SARS-CoV-2 pandemic. As of this writing, SARS-CoV-2 laboratory-confirmed cases have exceeded 147 million globally. Patients infected with this newly discovered coronavirus can be affected by either asymptomatic or symptomatic forms of the disease. The latter includes fever, sore throat, cough, shortness of breath, and a loss of smell and taste sensation. Some patients develop severe pneumonia and acute respiratory distress syndrome with fatal consequences (1). Older people and those with other health problems such as diabetes, cancer, lung diseases, and heart disease are the most vulnerable groups afflicted by the severe form of COVID-19. Early diagnosis and treatment of infected patients are important to avoid a complicated form of the disease and to prevent further spread of the virus to uninfected subjects (2).

Lymphopenia, elevated inflammatory markers such as high C-reactive protein (CRP), ferritin, and elevated D-dimer, in association with abnormal radiological findings in both chest X-rays and CT scans, are suggestive of COVID-19 (3). The COVID-19 Reporting and Data System (CO-RADS) is used as an indicator of the pulmonary engagement of COVID-19 (4). However, nucleic acid testing on respiratory tract specimens is the only laboratory confirmation of SARS-CoV-2. The angiotensin-converting enzyme 2 (ACE2) receptor has been recognized as the binding site of SARS-CoV-2 in the target cells (5). ACE2 plays a major role in patients with hypertension, CVD, obesity, and type 2 diabetes by opposing the harmful effects of the renin–angiotensin–aldosterone system (RAAS); therefore, the ACE/ACE2 ratio could be a good indicator of SARS-CoV-2 disease progression. There is a direct proportional relationship between COVID-19 infectivity and virulence that depends on the level of ACE2 expression on target cells (6, 7).

High-altitude-linked hypoxia has been reported to cause systemic inflammation associated with hypercoagulability and promotes venous thromboembolism development (8–10). Exposure to high altitudes has been reported to predispose patients to a series of thromboembolic events, due to increased blood hypercoagulability (11, 12). Platelet counts have been observed to rise within 1 week of exposure to high altitudes (13). Moreover, exposure to high altitudes for 1 year has been shown to increase plasma fibrinogen concentration by as much as 61% (14). In addition, a recent study showed that the mean platelet count is higher among high-altitude dwellers compared to low-altitude dwellers in Saudi Arabia, predisposing them for hypercoagulability (15).

The development of coagulation test abnormalities seen in COVID-19-infected patients is most likely a result of the profound inflammatory response caused by the virus and its associated coagulopathy (16). Based on these data, the combined causes of hypoxia and increased platelet counts in high-altitude dwellers may leave these people prone to more severe attacks of disseminated intravascular coagulation if they develop a COVID-19 infection. However, a recent study has reported less severe manifestations of the disease in people living in high-altitude regions (17).

The current study aimed to analyze the clinical characteristics, laboratory findings, risk factors, associated comorbidities, radiological findings, and outcomes in patients admitted to the hospital with a confirmed COVID-19 infection in Taif, Saudi Arabia. In addition, we also aimed to investigate the effect of high altitude on the severity of the disease, based on the clinical outcomes and disease severity in the hospitalized COVID-19 patients from one high-altitude city, Taif, and a sea-level city, Jeddah.

## Methods

### Patients and Time Frame

An observational retrospective study was conducted on a total of 760 COVID-19 laboratory-confirmed cases between April and September 2020 in Taif, Saudi Arabia. All patients were at least 18 years old and had confirmed positive polymerase chain reaction (PCR) results for COVID-19. Data from subjects were collected from the Alameen and King Faisal Hospitals, in Taif. An additional 208 subjects were recruited from Jeddah (which is at sea level), from patients at King Abdulaziz Hospital. The Jeddah data (*n* = 208) were compared to matched patients from Taif (*n* = 272/760). Matching patients' data from Taif were selected from the original 760 subjects based on patients' ages and comorbidities.

### Clinical and Laboratory Investigations

Initial investigations included complete blood count, highly sensitive CRP, and chest X-rays. CT chest scans were used as a routine investigation for most of the Taif patients during the early months of the epidemic. X-rays were performed on all patients, while the CT chest scans were only applied to 722/760 (95%) of the patients. Other investigations, such as D-dimer, ferritin, alanine aminotransferase, aspartate aminotransferase (AST), creatinine, international normalized ratio, prothrombin time, and partial thromboplastin time were also conducted. Supplemental oxygen therapy was used if patients' oxygen levels were <90%, to keep these levels between 92 and 96%. Patients who were admitted to an intensive care unit (ICU) were closely monitored for adequate hemodynamic support and fluid therapy, depending on both dynamic and static parameters. Patients who required supplemental oxygen were also given dexamethasone (6 mg daily), either orally or via intravenous injection.

### The Charlson Comorbidity Index

The relationships among the severity of COVID-19, ICU admission, disease outcome, and associated comorbidities were assessed in patients from Taif. Twenty-nine points were assigned to different comorbidities: a history of myocardial infarction (one point), congestive heart failure (one point), peripheral vascular disease (one point), dementia (one point), chronic pulmonary disease (one point), rheumatologic disease (one point), peptic ulcer disease (one point), liver disease (three points), diabetes (two points), hemiplegia or paraplegia (two points), renal disease (two points), malignancy (six points), and HIV/AIDS (six points). The Charlson comorbidity index was used as previously described (18–20).

### CO-RADS Classification

CO-RADS classification was used to classify patients from Taif according to their CT results (4). Those in the CO-RADS 1 group had a normal CT or showed non-infectious radiological changes, while those in CO-RADS 2 had a CT that showed changes relevant to non-COVID-19 infectious diseases. Those described as CO-RADS 3 showed an intermediate suspension of COVID-19, including widespread bronchopneumonia, lobar pneumonia, and ground-glass opacities (GGO) or septic emboli. Patients in CO-RADS 4 were cases in which COVID-19 was highly suspected. Unilateral GGO and multifocal consolidations without any other typical findings may be present in these patients' CT scans. A CO-RADS 5 classification is highly indicative for COVID-19, with CT showing multifocal GGO and consolidation. CO-RADS 6 was used to describe patients with a positive PCR with bilateral GGO.

### Statistical Analysis

Data are reported as means and standard error of mean (SEM) or counts and percentages, as appropriate. Comparisons among groups were made using *t* tests, the Mann–Whitney *U* test, chi-square, or Fisher exact tests, as dictated by data type and distribution. A multiple regression analysis using independent variables determined the predictors of ICU admission. One-way analysis of variance (ANOVA) was used to test differences among more than two groups. Pearson's or Spearman's correlations were used to test the correlations among variables. A *p* < 0.05 was considered significant for all statistical analyses in this study. All analyses were performed using the Statistical Package for the Social Sciences (SPSS) version 24 for Windows (SPSS, Inc., Chicago, IL, USA).

## Results

### Demographic Data of Patients From Taif

Healthcare workers accounted for 6.4% of all patients. The detected comorbidities included diabetes (16.2%), hypertension (12%), chronic obstructive pulmonary disease (3%), chronic kidney disease (2.6%), peripheral vascular disease (2.6%), coronary artery disease (1.8%), congestive heart failure (1.4%), and pulmonary embolism (PE) (3.8%). The symptoms included fever (84.7%), cough (58.2%), shortness of breath (45.3%), GIT symptoms (25.4%), sore throat (10.8%), anosmia (20.9%), and loss of taste sensation (15.7%). Sixty-four (8.5%) patients were admitted to the ICU, and 13 (1.7%) patients died. Patients were classified into three groups: (i) recovered patients without ICU admission, 695 (91.5%); (ii) recovered patients after ICU admission 52 (6.8%) patients; and (iii) deceased patients, 13 (1.7%) patients (Table 1).

**Table 1:** Clinical and demographic criteria of the studied groups of patients.

**Parameters**	**Total patients (760)**	**Recovered without ICU (695) (91.5%)**	**Recovered after ICU (1) (6.8%)**	**Passed away 13 (1.7%)**	***p***
**Age** mean (SEM) years	41.4 (0.53)	40.3 (0.6)^ab^	52 (2.3)^a^	57.3 (5.6)^b^	0.0001
**Gender**					
Male *n* (%)	578 (76.1)	530 (76.3)	40 (76.9)	8 (61.5)	0.468
Female *n* (%)	182 (23.9)	165 (23.7)	12 (23.1)	5 (38.5)	
**Nationality**					
Saudi *n* (%)	283 (37.2)	254 (36.5)	25 (48.1)	4 (30.8)	0.225
Others *n* (%)	477 (62.8)	441 (63.5)	27 (51.9)	9 (69.2)	
**Clinical presentation**					
Fever *n* (%)	644 (84.7)	590 (84.9)	42 (80.8)	12 (92.3)	0.58
Cough *n* (%)	442 (58.2)	401 (57.7)	33 (63.5)	8 (61.5)	0.735
Shortness of breath *n* (%)	344 (45.3)	312 (44.9)	24 (46.2)	8 (61.5)	0.492
GIT symptoms *n* (%)	193 (25.4)	182 (26.2)	8 (15.4)	3 (23.1)	0.227
Chronic diseases *n* (%)	197 (25.9)	155 (28.3)	31 (59.6)	11 (84.6)	0.0001
History of contact *n* (%)	178 (23.4)	171 (24.6)	6 (11.5)	1 (7.7)	0.041
Health care workers *n* (%)	49 (6.4)	47 (6.8)	1 (1.9)	1 (7.7)	0.354
Sore throat *n* (%)	82 (10.8)	79 (11.4)	1 (1.9)	2 (15.4)	0.046
Loss of smell sensation *n* (%)	159 (20.9)	153 (21)	5 (9.6)	1 (7.7)	0.046
Loss of taste sensation *n* (%)	119 (15.7)	116 (16.7)	3 (5.8)	0	0.033
**Associated co-morbidities and chronic diseases:**					
Hypertension *n* (%)	91 (14)	64 (9.2)	20 (38.5)	7 (53.8)	0.0001
DM *n* (%)	123 (16.2)	93 (13.4)	21 (40.4)	9 (69.2)	0.0001
CKD *n* (%)	20 (2.6)	11 (1.6)	5 (9.6)	4 (30.8)	0.0001
CLD *n* (%)	7 (0.9)	5 (0.7)	1 (1.9)	1 (7.7)	0.052
CHF *n* (%)	11 (1.4)	8 (1.2)	3 (5.8)	0	0.067
CVA *n* (%)	10 (1.3)	7 (2)	3 (5.8)	0	0.052
Peripheral vascular disease *n* (%)	20 (2.6)	10 (1.4)	7 (13.5)	3 (23.1)	0.0001
COPD *n* (%)	23 (4)	17 (2.4)	5 (9.6)	1 (7.7)	0.015
CAD *n* (%)	14 (1.8)	8 (1.2)	3 (5.8)	3 (23.1)	0.0001
Dementia *n* (%)	7 (0.9)	5 (0.7)	2 (3.8)	0	0.114
Rheumatological diseases *n* (%)	6 (0.8)	6 (0.9)	0	0	1
Peptic ulcers *n* (%)	23 (4)	21 (3%)	2 (3.8)	0	0.78
Malignancy *n* (%)	4 (0.5)	2 (0.3)	0	2 (15.4)	0.004
PE *n* (%)	29 (3.8)	7 (2)	12 (23.1)	10 (76.9)	0.0001
DVT *n* (%)	4 (0.5)	3 (0.4)	1 (1.9)	0	0.301
**Duration of admission (Days) (mean** **±** **SE)**	6.9 ± 0.23	6.2 ± 0.2^ab^	11.4 ± 1.6^a^	13.2 ± 2.1^b^	0.0001
**Charlson comorbidity score (mean** **±** **SE)**	1 ± 0.05	0.57 ± 0.05 ^ab^	1.7 ± 0.3 ^a^	3.9 ± 0.9 ^b^	0.0001

*Means followed by the same superscript is not statistically significant*.

Age is a major factor in the severity of COVID-19, as greater ages were associated with increased ICU admission and mortality rates, with *p* < 0.0001. We found no significant differences between the patient subgroups for gender and nationality. Patients who were admitted to the ICU and/or who died showed significant histories of hypertension, diabetes, chronic kidney disease, peripheral vascular disease, and coronary artery disease, with a *p* value of 0.0001 for all. We observed no significant differences in most of the initial clinical presentations among the patient subgroups, though patients with a history of ICU admission had a longer duration of hospital length of stay, with a *p* value of < 0.0001. Charlson's comorbidity scores were significantly higher in patients admitted to the ICU and/or who died, with a *p* < 0.0001 (Table 1).

Patients' mean oxygen saturation percentage at the time of admission was 94.6%, and 45.1% of our patients showed absolute lymphocytopenia. The mean level of high-sensitivity CRP was 23.6 (3.8) mg/L, the mean ferritin level was 540 (47.7) ng/ml, and the mean D-dimer level was 0.7 (0.1) mcg/ml. Patients with a history of ICU admission and/or death showed significant hypoxia, with a *p* < 0.001. Leukocytosis and neutrophilia were found to be significant among patients admitted to the ICU and/or who died, in addition to significantly higher D-dimer, ferritin, and highly sensitive CRP levels, with a *p* < 0.0001 for all patients (Table 2).

**Table 2:** Laboratory characteristic of the studied groups of patients.

**Item**	**Total patients**	**Recovered without ICU**	**Recovered after ICU**	**Passed Away**	***p***
Number	760	695 (91.5%)	52 (6.8%)	13 (1.7%)	
O_2_ saturation mean (%) (SEM)	94.6 (0.13)	94.8 (0.14)^ab^	90.6 (0.43)^ac^	85.2 (2.4)^bc^	0.0001
Total WBCs count mean (SEM)/ μl	7.1 (0.13)	6.8 (0.11) ^ab^	8.3 (0.6) ^ac^	12.8 (1.6) ^bc^	0.0001
Neutrophil count mean (SEM)/ μl	4.9 (0.11)	4.65 (0.1)	6.1 (0.5)	9.9 (1.4)	0.0001
Lymphocyte count mean (SEM)/ μl	1.7 (0.04)	1.7 (0.04)	1.5 (0.1)	1.75 (0.3)	0.186
Hb mean (SEM)gm/dl	14.2 (0.23)	14.4 (0.3)	13.8 (0.5)	13.5 (0.5)	0.137
PLT mean (SEM) μl	278.2 (10.4)	284.9 (13.5)	270.8 (17.6)	246.5 (27.3)	0.671
INR mean (SEM)	1.04 (0.01)	1.05 (0.01)	0.97 (0.02)	1.1 (0.05)	0.283
PT mean (SEM)/sec.	13.6 (0.12)	13.7 (0.13)	13.1 (0.19)	14.45 (0.6)	0.563
PTT mean (SEM)/sec.	35.6 (0.7)	35.5 (0.6)	35.2 (1.03)	36.6 (5.5)	0.903
D-Dimer CRP mean (SEM) mcg/mL	0.7 (0.1)	0.48 (0.06)^ab^	0.34 (0.05)^ac^	2.5 (0.88)^bc^	0.0001
High CRP mean (SEM) mg/L	23.6 (3.8)	10.7 (2.1) ^ab^	48.3 (13.1)^a^	65.7 (14.9)^b^	0.0001
Ferritin mean (SEM) ng/ml	540 (47.7)	416 (44.7)^ab^	745.9 (117.4)^a^	1003 (197.4)^b^	0.0001
ALT mean (SEM) IU/L	39 (5.4)	30.4 (2.7)	64.8 (25.5)	44.2 (7.9)	0.08
AST mean (SEM) IU/L	39 (5.1)	29.2 (1.8)	58.4 (23.2)	67.8 (15.9)	0.0001
Creatinine mean (SEM) μmol/L	78.2 (3.6)	78.4 (4.1)	102.75 (17.8)	70 (8.3)	0.258

*Means followed by the same superscript is not statistically significant*.

**Figure 1:**
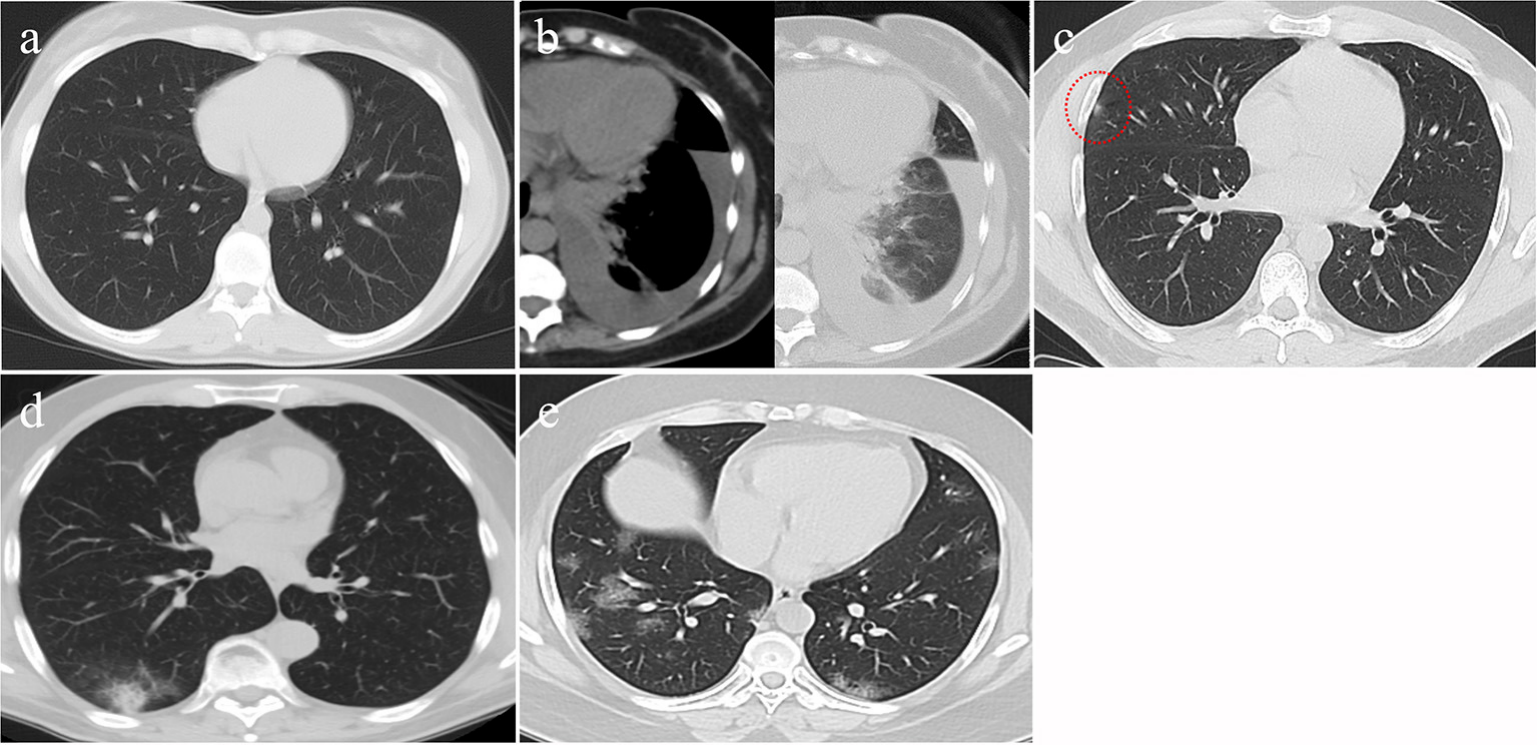
CT findings of different CO-RAD stages. **(a)** Normal CT findings, in keeping with CO-RAD1. **(b)** CT showed a left lower lobar patchy area of consolidation associated with moderate pleural effusion. This picture is suggestive of bacterial pneumonia (in keeping with CO-RAD2). Note that pleural effusion is very unlikely to occur with COVID-19 pneumonia. **(c)** A very tiny right middle lobar patchy area of faint ground glass opacity (red dashed circle), in keeping with CO-RAD3. **(d)** CT chest showed right lower lobar patchy area of ground glass veiling, mounting to consolidation, in keeping with CO-RADS 4. **(e)** Bilateral, mainly peripheral and lower lobar, small patchy areas of ground glass opacity, in keeping with CO-RADS 5.

A logistic regression analysis for patients admitted to an ICU showed that the level of highly sensitive CRP was the most independent predictor for death, with a *p* value of 0.003 (Table 3). Radiological investigations of our confirmed cases showed that 74.5% had positive results in a chest x-ray. By contrast, 95% of our patients had CT chest affections and were classified according to the CO-RADS classification system (Figure 1); 18.2% were CO-RADS 1, 3.8% were CO-RADS 2, 1.6% were CO-RADS 3, 2.9% were CO-RADS 4, and 68.6% were CO-RADS 5. There was a significant difference among patient subgroups regarding the chest CT and chest X-ray results, with *p*-values of 0.03 and 0.0001, respectively (Table 4).

**Table 3:** Logistic regression analysis for independent predictors of ICU admission.

**Variable**	**Sig**.	**Exp (B)**	**95% C.I. for EXP(B)**	
			**Lower**	**Upper**
Age	0.059	1.046	0.998	1.095
DM	0.663	0.806	0.305	2.129
HTN	0.646	0.666	0.117	3.781
CKD	0.855	1.196	0.176	8.139
CAD	0.597	0.325	0.005	20.944
Charlson comorbidity index	0.316	1.356	0.747	2.460
WBCS	0.714	1.105	0.649	1.880
Neutrophil	0.618	1.162	0.643	2.101
CRP	0.003	1.027	1.010	1.045
D-dimer	0.285	1.265	0.822	1.949
Ferritin	0.518	1.000	0.999	1.002
Constant	0.000	0.002		

**Table 4:** Radiological characteristics of the studied groups of patients.

**Item**	**Total patients**	**Recovered without ICU**	**Recovered after ICU**	**Passed Away**	***p***
Number (%)	760 (100%)	695 (91.5%)	52 (6.8%)	13 (1.7%)	
**X-ray findings**					
Positive *n* (%)	566 (74.5)	504 (72.5)	49 (94.2)	13 (100)	0.0001
**CT findings**					
CO-RADS 1 *n* (%)	138 (18.2)	129 (18.6)	9 (17.3)	0	0.03
CO-RADS 2 *n* (%)	29 (3.8)	29 (4.2)	0	0	
CO-RADS 3 *n* (%)	12 (1.6)	12 (1.7)	0	0	
CO-RADS 4 *n* (%)	22 (2.9)	20 (2.9)	1 (1.9)	1 (7.7)	
CO-RADS 5 *n* (%)	521 (68.6)	468 (67.3)	51 (98.1)	12 (92.3)	
Un-available *n* (%)	38 (6)	37 (5.3)	1 (1.9)	0	

Pearson's correlation was conducted between the different parameters with disease severity and radiological findings. Items that only showed significant correlation was presented in Table 5. Age, AST, D-dimer, ferritin, and CRP showed highly significant correlation (*P* > 0.01) with both disease severity and radiological findings.

**Table 5:** Correlation between clinical, laboratory, disease severity and CT findings.

**Item**		**Disease severity**	**CT results**
Age	*r*	0.249^**^	0.164^**^
	Sig.	0.000	0.000
Disease severity	*r*	1	0.080^*^
	Sig.		0.031
O_2_	*r*	−0.197-^**^	−0.039-
	Sig.	0.000	0.294
RBCs	*r*	−0.105-^*^	−0.069-
	Sig.	0.012	0.108
ESR	*r*	0.334^**^	0.016
	Sig.	0.000	0.763
HCT	*r*	−0.102-^*^	−0.066-
	Sig.	0.016	0.128
MCV	*r*	0.110^**^	0.026
	Sig.	0.009	0.553
WBCs	*r*	0.275^**^	0.000
	Sig.	0.000	0.995
Neutrophils	*r*	0.319^**^	0.041
	Sig.	0.000	0.337
Lymphocyte	*r*	−0.047-	−0.091-^*^
	Sig.	0.252	0.031
ALT	*r*	0.110^**^	0.084^*^
	Sig.	0.005	0.035
AST	*r*	0.221^**^	0.122^**^
	Sig.	0.000	0.002
Urea	*r*	0.118^*^	−0.103-^*^
	Sig.	0.013	0.033
D-dimer	*r*	0.225^**^	−0.005-
	Sig.	0.000	0.921
Ferritin	*r*	0.292^**^	0.163^**^
	Sig.	0.000	0.001
CRP	*r*	0.348^**^	0.221^**^
	Sig.	0.000	0.000

* *Correlation is significant at the 0.05 level*.

** *Correlation is significant at the 0.01 level*.

### Comparison of Matched Patients in High Altitude and Sea Level

Upon comparing the clinical data of matched patients from Taif and Jeddah, we noted that SARS-CoV-2 infection rates were found to be significantly higher in males than in females, and among subjects between the ages of 21–40 years and 41–60 years in both the high-altitude (Taif) and sea-level (Jeddah) regions (Table 6).

**Table 6:** Demographic data of Jeddah and Taif COVID-19 patients.

**Demographic factors**	**Categories**	**Jeddah**		**Taif**	
		**No**.	**%**	**No**.	**%**
Gender	Male	136	65.40	183	67.30
	Female	72	34.60	89	32.70
	Total	208	100.0	272	100.0
Age (year)	18–20	11	5.3	19	7.0
	21–40	104	50.0	132	48.5
	41–60	75	36.1	94	34.6
	61–80	14	6.7	25	9.2
	81+	4	1.9	2	0.7
Healthcare worker		16	7.69	20	7.35

Interestingly, we detected a significantly higher percentage of recovery in the high-altitude region, in comparison with those living at sea level. Importantly, the death rate in the sea level region was nearly threefold that reported in the high-altitude region (Table 7).

**Table 7:** Clinical, laboratory and deaths among COVID-19 ICU patients in Jeddah and Taif.

**Variable**		**Jeddah** **(*****n*** ***=*** **208)**		**Taif** **(*****n*** ***=*** **272)**		**χ**^**2**^	***P-value***
		**No**.	**%**	**No**.	**%**	
Fever	No	87	41.83	154	56.62	10.31	0.00^**^
	Yes	121	58.17	118	43.38		
Cough	No	98	47.12	163	59.93	7.79	0.00^**^
	Yes	110	52.88	109	40.07		
Shortness of breath	No	115	55.29	192	70.59	11.96	0.00^**^
	Yes	93	44.71	80	29.41		
Headache and sore throat	No	184	88.46	243	89.34	0.09	0.76
	Yes	24	11.54	29	10.66		
Smell	No	197	94.70	252	92.60	0.83	0.36
	Yes	11	5.30	20	7.40		
Loss of Taste	No	203	97.60	271	99.63	3.96	0.04^*^
	Yes	5	2.40	1	0.37		
GIT symptoms	No	181	87.02	233	85.7	0.183	0.669
	Yes	27	12.98	39	14.3		
Chronic diseases	No	175	84.13	238	87.5	1.11	0.29
	Yes	33	15.87	34	12.5		
Diabetes mellites	No	162	77.88	219	80.5	0.498	0.48
	Yes	46	22.12	53	19.5		
Hypertension	No	169	81.20	225	82.70	0.17	0.67
	Yes	39	18.80	47	17.30		
Asthma	No	194	93.30	251	92.30	0.17	0.67
	Yes	14	6.70	21	7.70		
DVT	No	205	98.60	256	94.10	6.11	0.01 ^*^
	Yes	3	1.40	16	5.90		
PE	No	205	98.60	244	89.70	15.28	0.00^**^
	Yes	3	1.40	28	10.30		
MI	No	206	99.00	259	95.20	5.67	0.01^*^
	Yes	2	1.00	13	4.80		
Ischemic stroke	No	202	97.10	259	95.20	1.11	0.29
	Yes	6	2.90	13	4.80		
ARDS	No	186	89.42	258	94.85	5	0.02^*^
	Yes	22	10.58	14	5.15		
Acute large vessels occlusion	No	207	99.52	259	95.22	7.69	0.00^**^
	Yes	1	0.48	13	4.78		
Coronary disease	No	203	97.60	260	95.59	1.39	0.23
	Yes	5	2.40	12	4.41		
Tumor	No	205	98.56	272	205	3.94	0.04^*^
	Yes	3	1.44	0	0		
CKD	No	203	97.60	270	99.26	2.28	0.13
	Yes	5	2.40	2	0.74		
Death	No	30	14.42	13	4.78	11.37	0.00^**^
	Yes	178	85.58	259	95.22		

* *P-value refers to statistically significant result*.

** *P-value refers to statistically highly significant result*.

Contrary to the above results, significantly higher rates of deep vein thrombosis, PE, MI, and acute large vessel occlusion were reported in the high-altitude region, compared to the sea-level region. However, the incidence of fever, cough, shortness of breath, and acute respiratory distress syndrome (ARDS) during the course of the disease were higher in Jeddah than in Taif. Importantly, the incidence of comorbidities such as diabetes mellitus, hypertension, asthma, ischemic stroke, and coronary disease showed no significant differences between the high-altitude and sea-level regions (Table 7).

## Discussion

In our study, as reported in other studies, patient age was a critical risk factor for ICU admission, with elderly patients being the most frequently admitted. In addition, comorbidities such as hypertension, diabetes mellitus, chronic kidney disease, ischemic heart disease, peripheral vascular disease, and malignancies were found to correlate with an increased probability of ICU admission and fatal consequences. Our findings also agree with previously available studies in this respect (21–25).

Patients who were admitted to the ICU and those who died showed significantly higher Charlson comorbidity scores than other patients did. These results were similar to previously published data from Denmark (26). Our results indicated that the Charlson comorbidity index is a valid indicator for COVID-19 patients with poor outcomes, in concordance with previous studies (27, 28).

In this study, we found no significant relationship between the presence of fever, cough, shortness of breath, GIT symptoms, and being a healthcare worker or with ICU admission or mortality. Our results did not match other studies (29, 30) that found a high fever to be associated with COVID-19 progression; we attribute this distance to differences in the studies' designs.

In contrast to the above, sore throat and a loss of smell and taste sensation were associated with a good prognosis among confirmed cases of COVID-19. Our results agree with a previous study that reported a correlation between smell loss and decreased hospitalization, intubation, and acute respiratory distress syndrome (31). In addition, another study also found that patients with a sore throat had better outcomes than others (32). However, there is no available explanation for these correlations.

Our study showed that low oxygen saturation at the time of admission was associated with poor outcomes. Hypoxia was found to be a powerful independent predictor of mortality in COVID-19 patients (33). Previous studies showed a strong relationship between hypoxia and both increasing the inflammatory response (34) and induction of viral replication (35, 36). It might also play a role in the pathogenesis of hypercoagulation (37).

In our study, we found that the presence of leukocytosis, neutrophilia, high levels of highly sensitive CRP, ferritin, D-dimer, and AST at the time of admission were associated with patients' deterioration, ICU admission, and death. Additionally, a significant correlation was found between such parameters and both the disease severity. These results were in line with previous studies that reported a significant association between disease progression and poor outcomes, with increased levels of inflammatory biomarkers and leukocytosis (38–40).

Logistic regression analysis showed that highly sensitive CRP was the most significant independent predictor of death among COVID-19 patients who were admitted to an ICU. This finding corresponds with previous reports that confirmed that CRP is the major determinant of disease severity in COVID-19 patients (41, 42). It may also highlight the role of secondary infection in the deterioration of patients' health status. Interestingly, the use of steroids was reported to be associated with secondary infection (43, 44).

In all, 18.2% of our patients showed normal chest CT appearance. Chest CTs were conducted early for most of our patients, and it is well-known that chest CTs continue to be normal in the first 4–5 days of infection (45). Meanwhile, among those who were admitted to the ICU and who later died, the chest CT results were significantly positive. At present, chest CT is not routinely ordered, and its use is limited to specific cases, especially in patients with false negative PCR (46, 47). However, we found a significant correlation of the radiological findings and the severity of the disease. This finding was also in accordance with recent findings that found similar correlation (48, 49).

We observed a lower mortality rate among people living at a high altitude, in comparison to those living at sea level. Another study has suggested a reduced severity of SARS-CoV-2 in high-altitude-dwelling patients (17); however, there are no current data explaining the mechanism involved in such an effect. The number of ACE2 receptors in target cells affects the susceptibility of such cells to SARS-CoV-2 infection. One speculation is the assumption that high-altitude inhabitants possess hypoxia-induced downregulation of ACE2. The RAAS is the expression of both ACE1 and ACE2 in negative feedback manner. During hypoxia, ACE2 is downregulated in the concomitant upregulation of ACE1 (50). Accordingly, decreased expressions of ACE2 in the pulmonary endothelial lining among high-altitude inhabitants is assumed to reduce the amplitude of virus replication (17). This speculation was argued strongly by Pun et al. (51), who presented conflicting results regarding ACE2. Their argument was augmented by many previous studies that reported the upregulation of ACE2 via hypoxia (52–54). Furthermore, deciphering hypoxia as a potential factor that alters the ACE2 expression is still obscure and needs further investigation. It is possible that some other, still-unknown mechanism renders high-altitude inhabitants more resistant to the severe consequences of COVID-19, compared to their sea-level-dwelling contemporaries.

## Conclusion

Fever, cough, and shortness of breath are the main clinical presentations in COVID-19 patients. Age, multiple comorbidities, a high Charlson comorbidity score, leukocytosis, neutrophilia, higher levels of highly sensitive CRP, ferritin, D-dimer, and AST are associated with poor outcomes among COVID-19 patients. High-altitude inhabitants showed reduced disease severity, in comparison with sea-level-dwelling patients.

## Data Availability Statement

The original contributions presented in the study are included in the article/supplementary material, further inquiries can be directed to the corresponding author/s.

## Ethics Statement

The studies involving human participants were reviewed and approved by Research and Studies Department, Directorate of Health Affairs, Taif, Saudi Arabia (NO. 397 on 23/07/2020). The patients/participants provided their written informed consent to participate in this study.

## Author Contributions

AF, AR, MR, RMMA, and AAA collected and analyzed the data from Alamin hospital. RMA, SAA, TA, and SMA collected and analyzed data from Jeddah. MA, ZI, DN, ANA, and DA analyzed the data and prepared the initial draft. DN, DA, ASA-M and AAA conceived the study protocol and critically revised the manuscript. All authors shared in data analysis and discussing and approved the final form of the manuscript.

## Conflict of Interest

The authors declare that the research was conducted in the absence of any commercial or financial relationships that could be construed as a potential conflict of interest.
